# Climate-driven sympatry may not lead to foraging competition between congeneric top-predators

**DOI:** 10.1038/srep18820

**Published:** 2016-01-06

**Authors:** Megan A. Cimino, Mark A. Moline, William R. Fraser, Donna L. Patterson-Fraser, Matthew J. Oliver

**Affiliations:** 1 College of Earth Ocean and Environment, University of Delaware, 700 Pilottown Rd., Lewes, DE 19958, USA; 2 Polar Oceans Research Group, Post Office Box 368, Sheridan, MT 59749, USA

## Abstract

Climate-driven sympatry may lead to competition for food resources between species. Rapid warming in the West Antarctic Peninsula (WAP) is coincident with increasing gentoo penguin and decreasing Adélie penguin populations, suggesting that competition for food may exacerbate the Adélie penguin decline. On fine scales, we tested for foraging competition between these species during the chick-rearing period by comparing their foraging behaviors with the distribution of their prey, Antarctic krill. We detected krill aggregations within the horizontal and vertical foraging ranges of Adélie and gentoo penguins, and found that krill selected for habitats that balance the need to consume food and avoid predation. In overlapping Adélie and gentoo penguin foraging areas, four gentoo penguins switched foraging behavior by foraging at deeper depths, a strategy which limits competition with Adélie penguins. This suggests that climate-driven sympatry does not necessarily result in competitive exclusion of Adélie penguins by gentoo penguins. Contrary to a recent theory, which suggests that increased competition for krill is one of the major drivers of Adélie penguin population declines, we suggest that declines in Adélie penguins along the WAP are more likely due to direct and indirect climate impacts on their life histories.

Climate directly influences species by affecting their physiology and life history^1^. Species’ distributions can be indirectly affected by new biotic interactions between species; for example, climate-driven sympatry can lead to niche displacement through competitive interactions^2^,^3^,^4^. Along the West Antarctic Peninsula (WAP), a climate migration from polar to subpolar conditions decreased the sea ice extent and coverage duration^5^ and altered the food web^6^. Coincidently, there has been an abrupt decline in the ice-obligate Adélie penguin (*Pygoscelis adeliae*) breeding population and an abrupt increase in the ice-intolerant gentoo penguin (*P. papua*) breeding population around Palmer Station, Anvers Island, WAP^7^. The competitive exclusion principle suggests that Adélie penguins could be displaced if they compete with gentoo penguins for the same food resources^8^. A recent hypothesis suggested that the decrease in Adélie penguin abundance in the WAP is due to increased competition between krill predators and a long-term decline in Antarctic krill (*Euphausia superba*), the penguins’ main prey^9^. If competitive interactions typically govern species distributions, climate-based projections of penguin populations will not be informative^10^. Therefore, rapid climate change along the WAP provides a unique opportunity to study the outcome of new climate-driven sympatry and to evaluate the importance of interspecific competition for common resources in a rapidly changing polar marine system.

Species competing for the same resources can coexist if they occupy different niches and find adequate resources within their niche^11^. In long-established colonies, previous studies demonstrate that penguin species avoid competition by using different foraging habitats horizontally^12^, vertically^13^, and temporally^14^. However, it is not clear if habitat partitioning occurs for newly established sympatric interactions due to recent climate changes. Although conspecifics or heterospecifics may occupy different niches with minimal foraging overlap, competition may still exist if the prey are highly mobile or sparse^12^. Antarctic krill are highly mobile because they are capable of directed movements over small^15^ and large spatial scales^16^, and are rapidly transported by winds^17^ and oceanographic conditions^18^. Therefore, studying penguin foraging behavior and the prey distribution within the foraging range is vital to identify resource competition between penguin species.

Penguins do not experience or feed on the average concentration of prey in their foraging domain, but rather use directed searching and different foraging behaviors to find rare, high concentrations of prey in the environment^19^. Therefore, to understand the marine environment as a penguin experiences it, it is necessary to sample on similar spatiotemporal scales of a foraging penguin. Traditional sampling methods using net tows or profiling equipment do not typically provide nearshore, concurrent, continuous, and high-resolution oceanographic data of multiple factors. However, autonomous underwater vehicles (AUVs), travelling at similar speeds, depth ranges and endurance as a foraging penguin, can sample the dynamic marine environment as experienced by a penguin^20^,^21^. These AUV’s navigate in nearshore coastal environments, and can simultaneously measure multiple trophic levels and physical properties of the water column. Near Palmer Station, congeneric Adélie and gentoo penguins are central place foragers and breed synchronously on nearby colonies during the austral summer. We focused on the chick-feeding phase of the breeding cycle when adults are provisioning chicks and parental foraging ranges of both species overlap. This period is a critical time for chick growth as fledglings with a higher body mass are more likely to survive^22^. We deployed an AUV informed by real-time positions of foraging penguins outfitted with satellite transmitters to measure the prey field and ocean properties. Using these innovative methods, we investigate the existence of competitive exclusion between sympatrically breeding Adélie and gentoo penguins.

## Results

### Habitat space of foraging penguins and prey aggregations

**Figure 1:**
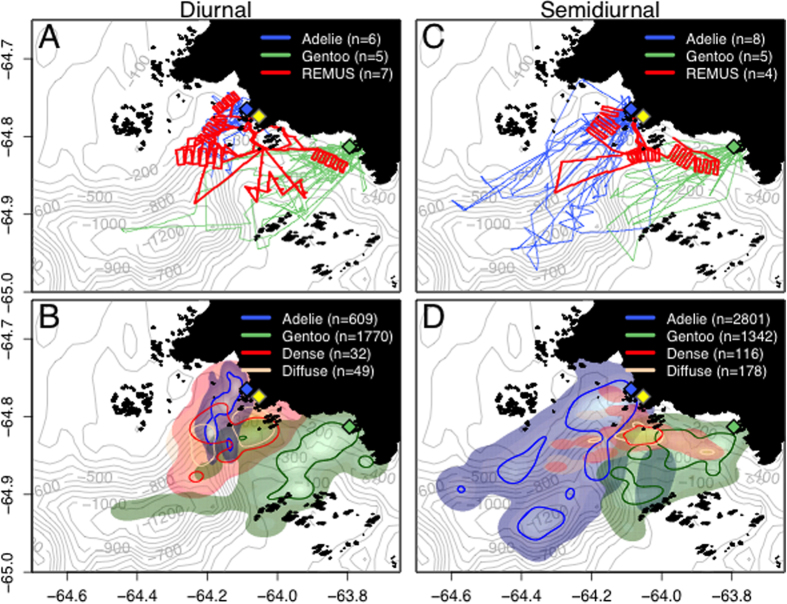
During diurnal and semidiurnal tides, the REMUS AUV detected prey aggregations within the foraging ranges of tagged penguins, near Palmer Station (yellow diamond), Antarctica. (**A**) The tracks of gentoo penguins breeding on Biscoe Point (green diamond) and Adélie penguins breeding on Humble Island (blue diamond), and areas sampled by the REMUS during 7 day-long missions during diurnal tides. (**B**) Kernel density estimates of foraging locations for Adélie and gentoo penguins, and dense and diffuse aggregations detected acoustically by the REMUS during diurnal tides. (**C**) Penguin tracks and areas sampled by the REMUS during 4 day-long mission and (**D**) the associated kernel density estimates during semidiurnal tides. The 50% contour lines (**B,D**) represent the core foraging areas of penguins, and the primary area with aggregation detections. For individual maps of kernel density estimates see Supplemental Fig. S1. The maps were produced in R (R Development Core Team 2014).

We studied the spatial habitat of foraging penguins and prey aggregations during diurnal and semidiurnal tidal regimes because it has been shown that tidal regime influences krill aggregation characteristics^18^ and Adélie penguin foraging location^23^. Adélie and gentoo penguins had spatially segregated foraging habitats (Fig. 1, Supplemental Fig. S1). Their core foraging areas, within the 50% contour of the kernel densities of foraging locations, were generally located near each of the penguin species’ respective colonies, with no foraging overlap between them (Fig. 1B,D). However, the area of the overlap in the overall foraging range was 17.18 km^2^ and 72.10 km^2^ during diurnal and semidiurnal tides, respectively. The Remote Environmental Monitoring UnitS (REMUS) AUV acoustically detected dense and diffuse aggregations within both species’ foraging ranges (Fig. 1B,D), which likely consisted mostly of densely and diffusely grouped krill, the dominant zooplankton species in the region and the driver of penguin foraging behavior. There were no differences between the depth, length or length-to-height ratio of dense or diffuse aggregations, but dense aggregations were significantly taller, larger in area and produced higher acoustic scattering (S_v_) within the aggregation (Table 1). These krill aggregation dimensions were similar to previous studies around Palmer Station^18^ and along the WAP^24^.

**Figure 2:**
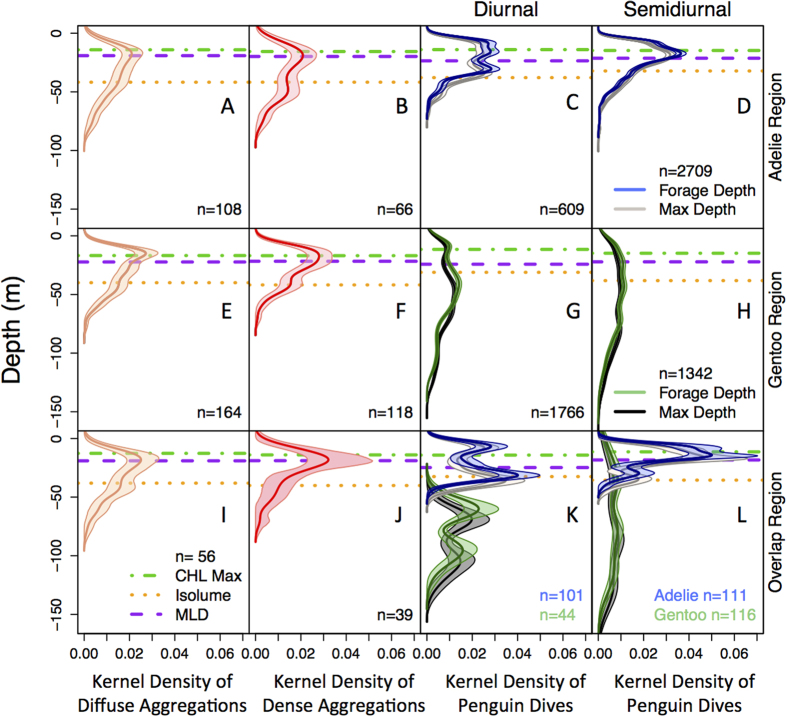
The vertical kernel density distribution of penguin dive and aggregation depths, and physical and biological properties of the water column within the Adélie penguin foraging habitat (top panel), gentoo penguin foraging habitat (middle panel) and the overlapping region where both species foraged (bottom panel) during diurnal and semidiurnal tides. The horizontal lines represent the mean depth of the CHL_max_, the 1 W/m^2^ isolume and mixed layer depth (MLD) within profiles with diffuse or dense aggregations and within each respective penguin foraging contour (Fig. 1B,D). (**A**) The kernel density estimate of the depth of diffuse and (**B**) dense aggregations within the Adélie foraging habitat that were measured during different tidal regimes were combined because there was no difference in their overall depth distributions between the tidal regimes (See Supplemental Fig. S2 for separation between tidal regimes). (**C**) The kernel density estimate of Adélie penguin foraging and maximum dive depths during diurnal and (**D**) semidiurnal tides. (**E**) The kernel density estimate of the depth of diffuse and (**F**) dense aggregations within the gentoo penguin foraging habitat during both tidal regimes. (**G**) The kernel density estimate of gentoo foraging and maximum dive depths during diurnal and (**H**) semidiurnal tides. (**I**) The kernel density estimate of the depth of diffuse and (**J)** dense aggregations within the overlapping region where both species forage during both tidal regimes. (**K**) The kernel density estimate of Adélie (n = 3) and gentoo penguin foraging (n = 2) and maximum dive depths within the region that both species utilized during diurnal tides, (**L**) the overlapping region between Biscoe Point and Humble Island during semidiurnal tides (Adélie n = 4, gentoo n = 2). The 95% confidence interval is shown around each kernel density estimate. Sample size (n) in each panel represents the number of aggregations or penguin dives.

In the upper 100 m of the water column, the depth distributions of diffuse and dense aggregations were generally very similar within different penguin foraging locations and tidal regimes. There were significantly deeper distributions of diffuse and dense aggregations in the Adélie penguin region than the gentoo penguin region (Supplemental Fig. S2A,C; K-S test, D = 0.46, p = 0.018; Supplemental Fig. S2B,D; K-S test, D = 0.47, p = 0.032). Additionally, within the Adélie penguin foraging region, dense aggregations were significantly deeper during diurnal tides than semidiurnal tides (Fig. 2B, K-S test, D = 0.43, p = 0.0078). Due to the small sample size of aggregation detections during diurnal tides (only ~20% of patch detections occurred during diurnal tides), it is difficult to determine if this was a real effect. Overall, the depth of all dense and diffuse aggregations was not affected by tides when ignoring location (LMM, Dense p = 0.51; Diffuse p = 0.61). Therefore, we grouped aggregations together between the two tidal regimes (Fig. 2). We found no significant differences between the depth distributions of dense and diffuse aggregations within the same foraging region or between diffuse/dense aggregations in different foraging regions. The depth of diffuse and dense aggregations peaked near the mixed layer depth (MLD) and chlorophyll maximum (CHL_max_) at ~20 m and there was often a secondary peak near the 1 W/m^2^ isolume at ~40 m.

**Figure 3:**
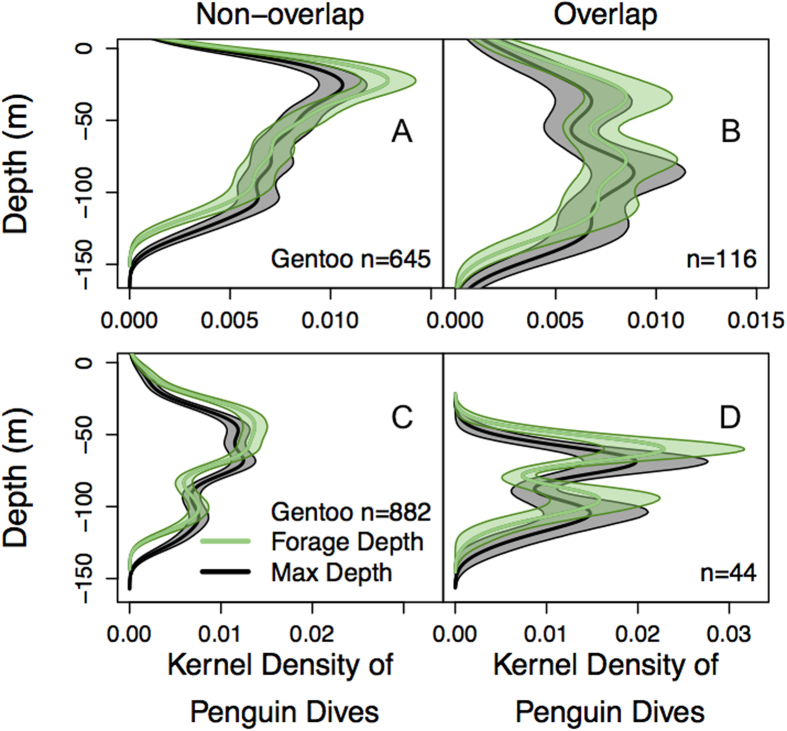
Comparison of gentoo penguin foraging dive depth distributions in areas that overlap and don’t overlap with Adélie penguins during diurnal and semidiurnal tides. (**A**) The kernel density estimate of gentoo penguin (n = 2) foraging and maximum dive depths in the area of non-overlap and (**B**) the area that overlaps with Adélie penguins during semidiurnal tides. (**C**) The kernel density estimate of gentoo penguin (n = 2) foraging and maximum dive depths in the area of non-overlap and (**D**) the area that overlaps with Adélie penguins during diurnal tides. The 95% confidence interval is shown around each kernel density estimate. Sample size (n) represents the number of penguin dives.

Penguin foraging dive distributions were also compared between tidal regime and location (Fig. 2). Notably, tidal regime had no affect on Adélie or gentoo penguin dive depths but there were some small differences in behavior during different tides. Sex had no affect on penguin dive depths or behavior. Gentoo penguin maximum dive depth was significantly deeper than Adélie penguins by 34.64% (Fig. 2C,D,G,H; LMM t-statistic = 5.31, p = 1.00 × 10^−4^). Generally, Adélie penguins did not dive below the average 1 W/m^2^ isolume (Fig. 2C,D) while gentoo penguin foraging and maximum dive depths were often below this depth (Fig. 2G,H). During diurnal and semidiurnal tides, four different gentoo penguin (2 per tidal regime) foraging ranges overlapped with those of Adélie penguins (Fig. 1C,D). In this area of overlap, gentoo penguins dove deeper than Adélie penguins during both tidal regimes (Fig. 2K; LMM t-statistic_max_depth_ = 7.74, p = 0.0045; t-statistic_forage_depth_ = 7.90, p = 0.0042; Fig. 2L t-statistic_max_depth_ = 3.73, p = 0.020; t-statistic_forage_depth_ = 3.35, p = 0.029). Additionally, in both cases, gentoo penguin foraging and maximum dive depths were significantly deeper in the area of overlap compared to the area of non-overlap (Fig. 3A,B, LMM t-statistic_max_depth_ = 5.00, p = 0; t-statistic_forage_depth_ = 4.85, p = 0; Fig. 3C,D; t-statistic_max_depth_ = 4.12, p = 0; t-statistic_forage_depth_ = 4.31, p = 0). During diurnal tides, one Adélie penguin entered the foraging domain of gentoo penguins (Fig. 1D). Here, the Adélie penguin foraging and maximum dive depths were not significantly different from gentoo penguins within the same region (Supplemental Fig. S3A) but the Adélie penguin dive depths were significantly deeper in the area of overlap compared to the area of non-overlap (Supplemental Fig. S3A,B, linear regression t-statistic_max_depth_ = 7.21, p = 5.95 × 10^−12^; t-statistic_forage_depth_ = 6.18, p = 1.71 × 10^−9^).

Adélie and gentoo penguins had similar and different proportions of foraging dive types. On average, 83.75 ± 8.07% of Adélie penguin foraging dives had bottom time, 16.70 ± 7.00% had vertical undulations or wiggles and 16.35 ± 10.10% had plateaus (ex. Supplementary Fig. S4). In comparison, 73.70 ± 8.06% of gentoo dives had bottom time, 27.75 ± 12.07% had wiggles and 16.73 ± 10.18% had plateaus. Using generalized linear mixed models (GLMMs), we found bottom time occurred less in gentoo foraging dives, and was positively related to the maximum dive depth (z_species_ = −3.55, p = −3.08 × 10^−4^; z_max_depth_ = −3.85, p = 1.12 × 10^−4^). There was no difference in the presence of wiggles between species but wiggles were more likely to be present in deeper dives (z_max_depth_ = 19.01, p = 2.60 × 10^−16^). Plateaus occurred more in the foraging dives of gentoo penguins than Adélie penguins, and were positively related to semidiurnal tides and negatively related to dive depth (z_species_ = 2.74, p = 6.24 × 10^−3^; z_max_depth_ = −21.54, p = 2.60 × 10^−16^; z_tide_ = 3.50, p = 4.67 × 10^−4^). The foraging dive durations of gentoo penguins were significantly longer than Adélie penguins by 39.23 s and foraging dive durations were significantly shorter during semidiurnal tides by 6.34 s (gentoo: 113.98 ± 14.70 s, Adélie: 74.75 ± 10.41 s, LMM t-statistic_species_ = 5.97, p = 0; t-statistic_tide_ = −3.44, p = 6.00 × 10^−4^), but Adélie penguins had a marginally higher dive frequency than gentoo penguins (Adélie: 17.67 ± 11.14 dives/hr, gentoo: 13.25 ± 7.24 dives/hr, LMM t = −2.05, p = 0.060).

### Presence/absence modeling of prey aggregations

We related the presence/absence of dense and diffuse aggregations to physical and biological properties associated with the vertical water column, all measured concurrently with the AUV (Supplemental Table S1). Nearly all of the models with substantial support (∆AIC < 2) showed the presence of dense and diffuse aggregations were associated with a deeper CHL_max_, lower integrated CHL and a shallower 1 W/m^2^ isolume. About half of these models showed a relationship between aggregation presence with a lower density at the MLD, which can be indicative of the strength of water column stratification and was related to a shallower MLD. Additionally, one or two models showed that aggregation presence was related to higher temperatures above the thermocline, deeper MLD, shallower thermocline, and lower surface photosynthetically available radiation (PAR). In general, the strongest predictor of both dense and diffuse aggregation presence was the 1 W/m^2^ isolume. These models were informative with an area under the curve (AUC) > 0.70 and with a moderately-high kappa value (~0.56), where a high kappa is >0.60. The percent correctly classified (PCC) was high (~92), but the models had low sensitivity (~50) and high specificity (~98). This suggests the models over predicted the number of absences. Notably, we found no significant differences between individual vertical profile characteristics associated with dense and diffuse aggregations from LMMs. This suggests the two aggregation types may be keying off similar water column properties, which our GLMMs also suggest.

Similarly, the mean depth of dense aggregations was positively related to depth of CHL_max_ (LMM t-stat = 1.98, p = 0.048), negatively related to surface PAR (LMM, t-stat = −2.22, p = 0.029), and marginally related to the isolume (LMM t-stat = 1.85, p = 0.067). The depth of diffuse aggregations was only marginally related to the density at MLD (LMM, t-stat = 1.74, p = 0.085).

## Discussion

Climate shifts can increase the intensity of sympatry between species and lead to interspecific competition, which has strong implications for understanding population trajectories and ecosystem structure^8^. In this study, we used a combination of penguin satellite telemetry, time-depth recorder and AUV data collected at the scale of the top predator to map the dynamic food resources used by both the Adélie and gentoo penguins. Sympatrically breeding Adélie and gentoo penguins had spatially segregated foraging habitats and mainly utilized the upper 100 m of the water column in regions of low interspecific competition (Fig. 2) but gentoo penguins foraged at deeper depths in areas of foraging overlap (Fig. 3). Subantarctic gentoo penguins, rather than Adélie penguins, switched behavior and vertically partitioned their foraging habitat to potentially avoid direct competition, even though shallow prey resources were available in the upper water column and both species are capable of deep dives. These species often employed different foraging strategies, which could be related to different interspecific dive strategies or prey aggregation characteristics. We detected two types of prey aggregations, dense and diffuse, at similar depths in the water column but without further study, it is unclear how these aggregation types influence penguin behavior. In the upper 100 m, the distribution of prey aggregations within penguin foraging regions were mainly associated with CHL and light and without observation of deeper prey aggregations, this could not explain the vertical segregation between species. Furthermore, both penguin species were provisioning chicks during satellite tracking, implying adults were returning to the nest with enough food, which also suggests that competition was not limiting food resources.

Krill are an important prey item in the Southern Ocean^25^ because they form large aggregations and have high nutritional value. Krill are a highly patchy and mobile resource; therefore, it is critical to study their distribution patterns to detect possible competition between top predators. We detected prey aggregations within the horizontal and upper vertical foraging ranges of Adélie and gentoo penguins (Figs 1 and 2). In contrast to^18^, we did not detect a large tidal effect on krill aggregation depth, which could be attributed to our observations being <100 m deep. We also did not detect differences in penguin dive depths between diurnal and semi-diurnal tides (Fig. 2), suggesting tide does not affect aggregation depths within the penguin foraging range. We detected fewer aggregations during diurnal tides, but with only 11 sampling days our inference is small. Aggregations were mainly found in areas that had a deeper CHL_max_, lower integrated CHL and shallower isolume (1 W/m^2^). Prey aggregations were likely selecting for habitats that balance the need to consume food and avoid predation, which is consistent with habitat choice theory^26^. Similar uncoupling between krill depth and chlorophyll was seen in the Ross Sea, which further highlights the trade-off between predator avoidance and food acquisition^21^. Light influences a species’ distribution because the pelagic environment offers few refuges from visual predators. High resource availability (i.e., high CHL) and predation are often correlated, suggesting high resource habitats are riskier^27^ and may lead to a negative relationship between aggregations and their food^28^. Each aggregation may select for different habitats based on individual variations in sex, age class, and conflicts between maximizing food intake and minimizing predation^26^,^29^. Nevertheless, our models suggest these aggregations had information about the availability of food or nearby ocean conditions^30^ and chose optimal habitats for increased survival.

Penguins are highly effective at finding food resources in a dynamic marine environment and can adjust their behavior to local foraging conditions^31^. Penguin foraging strategies can also change according to dietary preference, location, morphology, physiology, or oceanographic conditions, which may aid in explaining the different foraging dive types in Adélie and gentoo penguins. In our study, the main foraging dive type for Adélie and gentoo penguins was bottom time, but more Adélie penguin foraging dives had bottom time while more gentoo penguin foraging dives had plateaus. Plateaus may allow a penguin to silhouette its prey against a bright background when observed from below^32^ and attack prey with minimal warning. Bottom time in Adélie penguins is thought to be a successful strategy to obtain krill^33^. Gentoo penguins dove deeper and for longer durations, a likely result of their greater oxygen stores and body size^34^. Interestingly, near Palmer Station, Adélie penguins generally foraged in the upper 50 m (Fig. 2C,D) but are capable of much deeper dives to depths >150 m^35^ while gentoo penguins generally foraged in the upper 100 m but foraged as deep as 150 m (Fig. 2G,H). These species may have ideal foraging depths based on where they achieve neutral buoyancy, likely deeper for the larger-bodied gentoo penguin^36^, or preferences for how they attack krill aggregations (skimming off the top, mean aggregation depth, depth of maximum krill density, or behavioral strategies based on aggregation characteristics, etc.). The Adélie penguins’ small foraging range and shallow foraging depths suggest food resources were adequate in the nearshore waters around Palmer Station and they didn’t need to exploit deeper prey resources, or perhaps deeper prey were not equally available as in the gentoo penguin foraging region. For example, when one Adélie penguin entered the gentoo penguin foraging domain, it dove deeper in this area than when it did not overlap with gentoo penguins (Supplemental Fig. S3). This could indicate that shallower prey aggregations were less available or deeper prey aggregations are typically more available in the gentoo penguin foraging region, which could potentially explain the generally deeper foraging dives of gentoo penguins. Additional penguin foraging locations and prey observations below 100 m are necessary to decipher if differences in prey availability exist between the two species’ foraging domains. The one Adélie penguin foraging in the gentoo penguin domain was also likely competing for the same prey as gentoo penguins. This highlights the possible inflexibility of Adélie penguins to alter their behavior to buffer against direct competition.

Adélie and gentoo penguin foraging habitats were spatially and vertically segregated, which should minimize competition and allow for coexistence^37^. Competition was low near each species’ respective colony and both species utilized the upper water column (Fig. 2). It is more efficient to forage near the surface for air-breathing marine predators because it allows the time spent feeding to be maximized^38^. Interestingly, in regions of foraging overlap, gentoo penguins shifted their foraging dives to deeper depths below the foraging range of Adélie penguins (Figs 2K,L and 3). Here, the gentoo penguins utilized a habitat that was not utilized by Adélie penguins, which may be a successful behavioral strategy to acquire food. During this season, both species consumed krill of similar size classes (Supplemental Fig. S6) with <1% of their diet (wet mass weights) consisting of fish. Similar diets between species suggests they exploited the same prey field and diets did not change by foraging depth, which further supports that this change in behavior is not due to prey differences but rather, to buffer against competition. Admittedly, our sample size is small (four individuals), but all of the gentoo penguins in this study that entered the Adélie penguin foraging region displayed this behavioral switch (Fig. 3). This vertical segregation agrees with previous studies demonstrating spatiotemporal niche partitioning between gentoo and other *Pygoscelid* penguins^12^,^14^, and suggests this is a common strategy of gentoo penguins even in new sympatric interactions. The plasticity of gentoo penguin foraging behaviors to buffer against competition may be advantageous as climate changes and prey distributions are uncertain^39^.

Other factors could be important in driving and interpreting penguin foraging behavior in our study. Our sample size was small and it is possible that the foraging ranges and overlap between the two species would be larger if we tracked more individuals^40^ over a longer time period encompassing different breeding stages^41^. Although, the overall Adélie penguin spatial foraging distribution from 2002–2011^23^ is very similar to this study. This suggests that we are adequately representing the Adélie penguin foraging domain (Fig. 1) and tracking more individuals during the same breeding phase may not be more informative. Additionally, previous studies showed that larger penguin colonies had larger foraging areas, potentially due to the depletion of prey resources over time by more individuals^42^,^43^. Therefore, given the relatively small penguin breeding populations (<10,000 pairs) at Palmer Station, we may expect that the foraging range and overlap between species is also small. In addition, the availability of prey can be influenced by other physical factors. For example, the head of a large submarine canyon near Palmer Station (between the Adélie and gentoo penguin foraging regions) is recognized as an area of higher primary production that supports higher trophic levels^44^, but details on the role of the local topography on ocean currents, prey distributions and penguin foraging ecology is lacking.

Limited krill availability and competition from rebounding krill predators were hypothesized to explain the decline in Adélie penguin populations in the WAP and Scotia Sea^9^, but this study ignored increasing, krill-dependent, gentoo penguin populations in the same regions^45^. Furthermore, contrary to previous analyses showing large-scale krill declines along the WAP^6^, a new long-term krill study shows that krill populations are not declining in response to a changing WAP climate^46^. Taken with our analysis, if similar foraging segregation is a characteristic of WAP colonies, we hypothesize that the declining Adélie penguin populations in the WAP are unlikely solely driven by competition or krill limitation. Further research is needed to support this hypothesis but in light of recent studies, we want to point out that other possible drivers should not be ignored. Population trajectories may be affected by different life history strategies, foraging behavior or wintering habitats, which can be influenced by climate and sea ice conditions. Therefore, the “sea ice hypothesis” cannot be discounted due to the direct and indirect effects of sea ice on the availability of wintering habitats and krill^47^.

In a warming climate, it is expected that species better suited to a northern subantarctic climate will displace ice-obligates^1^. Our results suggest that foraging competition may not always cause displacement, especially for penguins that are known to partition their habitat with competitors or have the behavioral flexibility to cope with competition. Sympatrically breeding Adélie and gentoo penguins exhibited different foraging strategies and had segregated foraging habitats, suggesting that food resources are not limiting and competitive exclusion may not be a main driver of opposing population trends. Foraging competition needs to be compared between multiple years and information on deep prey aggregations is necessary to understand differences in dive depth distributions between species. Previous studies near Palmer Station demonstrate how climate and weather influence penguin breeding habitats^48^, the marine foraging environment^49^, foraging trip duration^50^ and chick mass^51^. We suggest future studies focus on wintering habitats and life history strategies, which are also likely affected by large-scale climate forcing. In particular, we must look outside the breeding season to fully understand ecological structure and the vulnerability of a species to dynamic marine environments. A balance of small and large-scale studies can provide insights into habitat use of keystone species, drivers of top predator population trends and ultimately, a synoptic understanding of the consequences of climate shifts in the Southern Ocean.

## Methods

### Penguin tracking and swimming behavior

From January 4 to 31, 2011, we studied Adélie and gentoo penguin foraging ecology near Palmer Station, Anvers Island, Antarctica (64° 46′ S, 64° 03′ W). We deployed satellite transmitters on Adélie penguins (5 female, 5 male) from Humble Island (64° 46′ S, 64° 06′ W) and gentoo penguins (5 female, 3 male) from Biscoe Point (64° 49′ S, 63° 46′ W) (Fig. 1A,C). All protocols were carried out in accordance with the approved guidelines of the Marine Biological Laboratory Institutional Animal Care and Use Committee, Assurance #A3070-01. The penguin location and depth data were filtered to remove inaccurate locations and corrected for drift in depth sensors (Supplementary Information). We classified penguin dive behaviors into transit, search and foraging dives (Supplementary Fig. S4). Foraging dives consisted of wiggles, plateaus or bottom time^52^ where penguins pursue or consume prey. The depth of foraging or most frequent depth was calculated using a kernel density estimate^53^. We determined the proportion of each foraging behavior (bottom time, wiggles or plateaus) for each individual and then each species (mean ± SD). We tested for differences in the presence and absence of different foraging behaviors in Adélie and gentoo penguins using GLMMs fit by maximum likelihood using glmer in the lme4 package^54^ in R (R Development Core Team 2014). Mixed models are useful when repeated measurements are made on an individual or related clusters because it takes the correlation of these repeated measures into account. Previous studies show that diurnal tides aggregate krill^18^ and correspond to shorter distance penguin foraging trips^23^. We accounted for a tidal effect on penguin foraging behavior by classifying each day as diurnal or semidiurnal^23^. We treated the individual as a random effect and included maximum dive depth, tide and sex as covariates. We used a binomial error structure because our dependent variable was the presence or absence of each behavior. We also tested for significant differences in foraging dive duration and dive frequency (number of dives/hr) using LMMs treating the individual as a random effect using the nlme package^55^. The residuals of the LMMs were normal. Results were considered to be statistically significant when p < 0.05 and marginally significant when 0.05 < p < 0.10.

### AUV data collection

A propeller-driven REMUS-100 AUV^20^ was deployed for 11 days between January 12 to 31, 2011, within the foraging regions of penguins near Palmer Station (Fig. 1A,C). The REMUS was equipped with sensors to measure temperature, density, CHL, PAR and relative acoustic backscatter (S_v_) (Supplementary Information). Zooplankton and fish are known to aggregate in groups of various densities, which has an unknown effect on predators’ acquisition of prey. To investigate differences between aggregation types, we identified dense aggregations from S_v_ measurements, likely consisting of densely grouped krill, fish and other zooplankton, and diffuse aggregations, likely consisting of less densely aggregated groups of zooplankton (Supplementary Information). Data from the project can be found here (http://gcmd.nasa.gov/getdif.htm?NSF-ANT10-19838).

The REMUS undulated in a seesaw pattern and we treated each vertical undulation as a vertical profile of the water column. We characterized each profile by determining the CHL_max_, integrated CHL in the upper 50 m, depth of the CHL_max_, MLD (the depth of the maximum change in density), density at the MLD, surface PAR (mean of the upper 2 m), depth of the 1 W/m^2^ isolume, thermocline depth, and the mean temperature above/below the thermocline. The depth of the CHL_max_ was not affected by non-photochemical quenching (NPQ)^56^, and integrated CHL was highly correlated to integrated CHL with regions affected by NPQ removed. We also created profiles of the background S_v_ by removing all aggregations and taking 1 m depth averages of S_v_ within 3 m of the REMUS. We found that profiles of background S_v_ were highly correlated to CHL profiles, which suggests that zooplankton were either highly coupled with CHL distributions or we were actually detecting larger chain-forming or aggregated diatoms (ex. Supplementary Fig. S5).

We tested for significant differences between physical and biological properties associated with dense and diffuse aggregations using a LMM. We treated each sampling day as a random effect in order to account for repeated measurements taken each day and for spatiotemporal variability. We also tested for relationships between the mean depths of aggregations, tide and water column properties using LMMs, treating day as a random effect. We square-root transformed mean depth to achieve normality.

### Presence/absence modeling of aggregations

We predicted the presence/absence of a dense or diffuse aggregation given water column properties. We used the information-theoretic model comparison (ITMC) approach to test multiple hypotheses, compare a suite of candidate models, and to select a small set of best approximating models^57^,^58^. We tested multiparameter candidate models using different combinations of explanatory variables, based on our hypotheses and existing knowledge on the system. We fit presence-absence GLMMs with glmer, included a random intercept term for sampling day and a binomial error structure. The Akaike Information Criteria (AIC) allowed us to choose the most parsimonious model that accounts for the most variation with the fewest terms, and we considered models with ∆AIC < 2 to have substantial support^59^. We tested for multicollinearity between predictor variables using variance inflation factors, but values < 4 indicated multicollinearity was not present in our models^60^. Prior to modeling, all predictor variables were standardized^61^.

To validate our models, we used the package PresenceAbsence^62^ to preform a 10-fold cross-validation resampling procedure. We repeated this procedure 10 times and calculated predictive accuracies with Cohens’s kappa statistic^63^, sensitivity (true positive rate), specificity (true negative rate), PCC, and AUC, which estimates receiver-operating characteristics^64^. The kappa statistic measured the proportion correctly classified after accounting for probability of chance agreement. The AUC is a measure of accuracy that is prevalence and threshold independent, and evaluates the false and true positive error rate^65^. An AUC of 1 represents perfect model performance and values below 0.5 are no better than random. To demonstrate model performance, we report the mean ± SD of 10 iterative runs from cross-validated estimates.

### Overlap between penguins and aggregations

We focused on the spatiotemporal overlap of foraging penguins and the detection of dense and diffuse aggregations (see Supplementary Table S2 for details on aggregation detections). The spatial region occupied by penguins and aggregations was determined using two-dimensional kernel density estimations with an axis-aligned bivariate normal kernel^66^ (Fig. 1B,D).

We compared the depth distributions of penguin dives and aggregations within Adélie and gentoo penguin foraging regions (Fig. 2). We used a two-sample Kolmogorov–Smirnov K-S) test, which is a nonparametric test that compares two one-dimensional probability distributions and quantifies the distance between two distribution functions^66^. We performed a two-sided K-S test to determine if there was a significant difference (p < 0.05) between distributions, if so we used an alternative K-S test to determine if a distribution was greater or less than the other distribution. We used a simpler K-S test instead of a complex mixed model because the Intra Class Correlations (ICCs) were low (<30%). ICC represents a measure of reliability or dependence among individuals^67^. A low ICC suggests there is a low correlation among observations within the same cluster and that no random effect is present in the data. Within the Adélie, gentoo, and overlapping foraging regions, we tested for a day effect on diffuse and dense aggregation depths. We used LMM to test for differences in penguin dive depths within different foraging regions. If necessary, dependent variables were log10 or square root transformed to achieve normality.

## Additional Information

**How to cite this article**: Cimino, M. A. *et al.* Climate-driven sympatry may not lead to foraging competition between congeneric top-predators. *Sci. Rep.* **6**, 18820; doi: 10.1038/srep18820 (2016).

## Supplementary Material

Supplementary Information

## Figures and Tables

**Table 1:** Differences in size and acoustic return between dense and diffuse aggregations detected by the REMUS AUV.

	Dense Aggregation	Diffuse Aggregation	Mann-Whitney U-test Z	Mann-Whitney U-test p-value
n	148	227		
Depth (m) (mean ± SD)	30.25 ± 16.24	30.91 ± 17.44	−0.19	0.85
Height (m)	5.91 ± 7.34	2.8 ± 2.61	−3.47	***0.00049***
Length (m)	40.44 ± 59.02	19.61 ± 21.09	−1.33	0.18
Area (m^2^)	198.14 ± 543.05	19.91 ± 20.2	−4.23	***2.02 *****×***** 10***^***−5***^
Length: Height ratio	6.58 ± 3.17	8.32 ± 8.34	1.65	0.099
S_v_ of aggregations	−61.90 ± 4.53	−66 ± 2.72	−12.62	**<*****2.2e-16***

We determined the depth, height (difference between the deepest and shallowest measurement), length (distance between the beginning and end of an aggregation), the ratio of length to height, and the area occupied by each aggregation. We tested for significant differences between dense and diffuse aggregation characteristics using a non-parametric Mann-Whitney U-test because the data was not normally distributed.
