# Supplementary material for: Climate-driven sympatry may not lead to foraging competition between congeneric top-predators
Source: Sci Rep. 2016 Jan 6;6:18820. doi: 10.1038/srep18820 (PMC4702144; doi:10.1038/srep18820)
Supplement: Supplementary Information [file srep18820-s1.doc]

Climate-driven sympatry may not lead to foraging competition between congeneric top-predators

Megan A. Cimino, Mark A. Moline, William R. Fraser, Donna L. Patterson-Fraser, Matthew J. Oliver

**SUPPORTING INFORMATION**

**Penguin satellite transmitter deployment details**

We instrumented penguins with satellite transmitters based on a custom SPLASH tag configuration (Wildlife Computers Redmond, WA, USA). Satellite transmitters had a pressure sensor to measure dive depths (resolution of 0.05m and accuracy of 2m) and dive data was recorded at 1 Hz. Penguins selected for instrumentation were paired and had brood-stage nests containing two chicks. Satellite transmitters were attached to the anterior body feathers using waterproof tape and small plastic cable ties. The transmitters had a sloped frontal area of 17×18 mm (306 mm^2^), weighed 55 g, had an antenna length of 12 cm and tag length of 86 mm. The transmitters represented < 2% of the penguins body mass and were rotated to new penguins every 3-5 days depending on weather conditions.

**Penguin dive behavior analysis**

The penguin location data were filtered following the methods of ^1^ to remove inaccurate location data due to erroneous terrestrial positions, unreasonable locations based on maximum sustained swimming speed (8 km/hr) ^2^ and coastal geometry. We corrected for drift in TDR depth sensors using recursive filtering and a diving threshold in the diveMove package ^3^ in R (R Development Core Team 2014). We time-matched dive records to location data and linearly interpolated between Argos locations to have an approximate location for all depth data. Penguin diving behavior varies between day and night ^4^. Therefore, we removed all dives that occurred during civil twilight (when the sun is less than 6º below the horizon) ^5^ because few dives occurred during this time and dives were generally shallower. In addition, the REMUS did not sample during civil twilight.

We classified penguin dives into transit, search and foraging dives (Supplementary Fig. S2). A transit dive is a near-surface traveling behavior with a duration < 20s ^6^ or a maximum depth < 5m ^7,8^. The dive duration was the time span between the start and end of the dive between the first and last surfacing ^9^.

Foraging dives were longer dives with wiggles, bottom time or plateaus (Supplementary Fig. S4). Wiggles are vertical undulations in the dive profile that reflect prey pursuit or encounters ^10,11^. A wiggle was defined as a deviation in depth > 2 m (error range on tag) with the vertical speed getting through 0 ^9,10^. The number of wiggles was likely underestimated because many wiggles had a vertical depth change of < 2m ^10^. Bottom time occurred during the flat part of a dive or near the maximum depth ^6,12^. Bottom time was the amount of time spent within 85% of the maximum depth (also known as the ledge) ^5,11,13^. We calculated the time spent below the ledge and considered foraging to occur if the time spent below the ledge was greater than 25% of the total dive duration. At depths shallower than the ledge, plateaus were events that were relatively long (temporal) and horizontal (spatial), and often consisted of wiggles ^14,15^. Plateaus can occur during the descent, ascent, or can be a long and horizontal phase between two deep diving periods ^14,15^. We defined a plateau as a period having an overall variation in depth of less than 10% of the maximum dive depth ^14^ and occurring for more than 25% of the dive duration. Previous studies have used a more lenient threshold of 10% ^14^ but we found a higher threshold to be more appropriate and allowed us to focus on dives where intense prey pursuit likely occurred. Foraging dives had bottom time, 2 or more wiggles ^16^, a plateau, or were > 90 seconds (6, 9).

V-shaped dives are typically search or exploratory dives, where the penguin does not spend a significant amount of time at any one depth. These dives consisted of no bottom time, < 2 wiggles, no plateaus and < 90 seconds. We calculated the penguins swimming speed (descent/ascent rate) as the change in depth divided by time. The average rate of descent (ROD) was calculated between the surface and the ledge (85% of the maximum depth). The average rate of ascent (ROA) was after the maximum depth and between the ledge and the surface. If bottom time was present, the average ROD had to be < 0.7m/s ^9^ to be considered a search dive. This threshold assumes that a foraging penguin dives at a steeper angle or greater ROD to a foraging depth compared to a traveling or searching penguin.

**Dense and diffuse aggregation detection**

The REMUS AUV missions ranged from 2 to 9 hrs (mean 6.8 ± 1.8 hr) where the REMUS traveled 13 to 54 km (mean 40.3 ± 10.7 km). The average speed was 1.6 ± 0.07 m/s. The REMUS undulated in a seesaw pattern between the surface and ~60m at a pitch of 6º, while occasionally pausing at the surface for a GPS fix. The REMUS was equipped with a Neil-Brown CTD, Wetlabs ECO-series backscatter/ fluorometer, Satlantic downwelling irradiance OCR-5071 sensor and Satlantic upwelling radiance OCR-507R sensor. The wavelengths for the OCR sensors are centered at 412, 442, 490, 532, 555, 669, and 683 nm and were converted into photosynthetically available radiation (PAR) from downwelling irradiance. Data from all these sensors were averaged into 1 m depth bins.

Acoustic backscatter was also measured using an upward- and downward-looking1200-kHz RD Instruments Workhorse Navigator acoustic Doppler current profilers (ADCP). The ADCPs provided relative acoustic backscatter (S_v_) as an estimate of scattering volume, instead of current velocities. The range limits on the ADCP were restricted to 8.75 m (upward-looking ADCP) and 6.75 m (downward-looking ADCP) due to high acoustic noise in the system. S_v_ measurements were binned vertically into 0.25m depth bins and horizontally into 8m bins. All acoustic measurements taken at depths < 5m were removed due to high surface noise and measurements from the downward-looking ADCP that could have resulted from bottom reflections were eliminated.

From acoustic measurements, we first identified dense aggregations that likely consisted of densely grouped krill, fish or other zooplankton. A running 5 m vertical median was taken for each day ^17^. The points at which a bin crossed above the daily median were used to define the upper and lower edges of the aggregation ^18^. The local background value was defined as the average value of these two crossing points ^18^. A bin was considered a dense aggregation when values of the bin exceeded 1.2 times the local background (18). The minimum size of a dense aggregation was a height of at least 1 m (4 vertical bins) or a width of ~16 m (2 horizontal bins). If an aggregation was simultaneously measured on the upward and downward looking ADCP, it was considered one aggregation. All aggregations were visually examined to confirm detection.

Krill, zooplankton and fish can also occur in less densely aggregated groups or layers, which would produce a lower acoustic return. To detect this type of diffuse aggregation, we removed all dense aggregations from the acoustic dataset. We calculated a new median and used the same methodology as described above to identify diffuse aggregations.

**REFERENCES**

1. Oliver, M. J. et al. Adelie Penguin Foraging Location Predicted by Tidal Regime Switching. *PLoS ONE* **8**, e55163 (2013).

2. Ainley, D. G. *The Adélie Penguin: Bellwether of Climate Change* (Columbia University

Press, New York, 2002).

3. Luque, S. P. Diving behaviour analysis in R. *R news* **7**, 8-14 (2007).

4. Miller, A. K., Kappes, M. A., Trivelpiece, S. G. & Trivelpiece, W. Z. Foraging-niche separation of breeding gentoo and chinstrap penguins, South Shetland Islands, Antarctica. *The Condor* **112**, 683-695 (2010).

5. Pütz, K. & Cherel, Y. The diving behaviour of brooding king penguins (*Aptenodytes patagonicus*) from the Falkland Islands: variation in dive profiles and synchronous underwater swimming provide new insights into their foraging strategies. *Mar. Biol.* **147**, 281-290 (2005).

6. Chappell, M. A., Shoemaker, V. H., Janes, D. N., Bucher, T. L. & Maloney, S. K. Diving behavior during foraging in breeding Adélie penguins. *Ecology* 1204-1215 (1993).

7. Kokubun, N., Takahashi, A., Mori, Y., Watanabe, S. & Shin, H. C. Comparison of diving behavior and foraging habitat use between chinstrap and gentoo penguins breeding in the South Shetland Islands, Antarctica. *Mar. Biol.* **157**, 811-825 (2010).

8. Takahashi, A. et al. Foraging strategies of chinstrap penguins at Signy Island, Antarctica: importance of benthic feeding on Antarctic krill. *Mar. Ecol. Prog. Ser.* **250**, 279-289 (2003).

9. Rodary, D., Wienecke, B. C. & Bost, C. A. Diving behaviour of Adélie penguins (*Pygoscelis adeliae*) at Dumont D’Urville, Antarctica: nocturnal patterns of diving and rapid adaptations to changes in sea-ice condition. *Polar Biol.* **23**, 113-120 (2000).

10. Bost, C.-A. et al. Changes in dive profiles as an indicator of feeding success in king and Adélie penguins. *Deep Sea Res. II* **54**, 248-255 (2007).

11. Kirkwood, R. & Robertson, G. The foraging ecology of female emperor penguins in winter. *Ecol. Monogr.* **67**, 155-176 (1997).

12. Wilson, R. P. in *Bird Families of the World: The penguins Spheniscidae* (eds Perrins, C., Bock, W. & Kikkawa, J.) 81-106 (Oxford University Press, Oxford, 1995).

13. Wienecke, B., Robertson, G., Kirkwood, R. & Lawton, K. Extreme dives by free-ranging emperor penguins. *Polar Biol.* **30**, 133-142 (2007).

14. Halsey, L. G., Bost, C.-A. & Handrich, Y. A thorough and quantified method for classifying seabird diving behaviour. *Polar Biol.* **30**, 991-1004 (2007).

15. Ropert-Coudert, Y. et al. Preliminary investigations of prey pursuit and capture by king penguins at sea. *Polar Biosci.* 101-112 (2000).

16. Wilson, R. P., Culik, B. M., Peters, G. & Bannasch, R. Diving behaviour of Gentoo penguins, *Pygoscelis papua*; factors keeping dive profiles in shape. *Mar. Biol.* **126**, 153-162 (1996).

17. Moline, M. A. et al. Integrated measurements of acoustical and optical thin layers II: Horizontal length scales. *Cont. Shelf Res.* **30**, 29-38 (2010).

18. Benoit-Bird, K. J., Moline, M. A., Waluk, C. M. & Robbins, I. C. Integrated measurements of acoustical and optical thin layers I: Vertical scales of association. *Cont. Shelf Res.* **30**, 17-28 (2010).

**Supplemental Table S1.** Summary of performance parameters for dense and diffuse aggregation presence-absence models using the information-theoretic model comparison approach. (AIC: Akaike Information Criterion, ∆AIC: difference from lowest AIC, Akaike weight representing relative model support or probabilities, AUC: area under the curve, PCC: percent correctly classified). Models with a substantial support (∆AIC < 2) are in bold. Variables in the models: CHLmax: chlorophyll maximum, depth_CHLmax: depth of the chlorophyll maximum, MLD: mixed layer depth, density_MLD: density at the mixed layer depth, isolume: 1 W/m2 isolume, surface_PAR: Photosynthetically available radiation at the surface, integrated_CHL: integrated chlorophyll above 50 m, thermocline: depth of greatest change in temperature, temp_above: mean temperature above the thermocline, temp_below: mean temperature below the thermocline)

|  | **Model** | **AIC** | **∆AIC** | **Akaike weight** | **AUC** | **PCC** | **Sensitivity** | **Specificity** | **Kappa** |
| --- | --- | --- | --- | --- | --- | --- | --- | --- | --- |
| ***Dense Aggregations*** | |  |  |  |  |  |  |  |  |
| 1 | **depth_CHLmax + density_MLD + integrated_chl + isolume** | 348.93 | **0.00** | 0.20 | 0.72 ± 0.0047 | 92.63 ± 0.18 | 46.07 ± 0.92 | 98.82 ± 0.15 | 0.56 ± 0.011 |
| 2 | **depth_CHLmax + density_MLD + MLD + integrated_chl + isolume** | 349.74 | **0.80** | 0.13 | 0.73 ± 0.0058 | 92.82 ± 0.23 | 46.4 ± 1.1 | 99 ± 0.14 | 0.57 ± 0.014 |
| 3 | **depth_CHLmax + density_MLD + MLD + integrated_chl + isolume + surface_PAR** | 350.14 | **1.21** | 0.11 | 0.73 ± 0.0035 | 92.68 ± 0.25 | 47.87 ± 0.58 | 98.64 ± 0.25 | 0.57 ± .012 |
| 4 | **depth_CHLmax + integrated_chl + isolume** | 350.31 | **1.38** | 0.10 | 0.72 ± 0.0036 | 92.48 ± 0.11 | 44.16 ± 0.76 | 98.91 ± 0.12 | 0.54 ± 0.0067 |
| 5 | **depth_CHLmax + integrated_chl + isolume + temp_above** | 350.42 | **1.49** | 0.09 | 0.72 ± 0.0049 | 92.51 ± 0.15 | 44.83 ± 0.98 | 98.85 ± 0.12 | 0.55 ± 0.0099 |
| 6 | **depth_CHLmax + density_MLD + temp_above + integrated_chl + isolume** | 350.45 | **1.52** | 0.09 | 0.72 ± 0.0036 | 92.72 ± 0.083 | 45.39 ± 0.79 | 99.01 ± 0.1 | 0.56 ± 0.0061 |
| 7 | depth_CHLmax + MLD + surface_PAR | 362.56 | 13.62 | 0.00 | 0.71 ± 0.0047 | 92.68 ± 0.19 | 42.47 ± 0.89 | 99.36 ± 0.14 | 0.54 ± 0.12 |
| 8 | depth_CHLmax + isolume | 364.19 | 15.26 | 0.00 | 0.71 ± 0.0024 | 92.64 ± 0.056 | 41.8 ± 0.47 | 99.4 ± 0 | 0.54 ± 0.0047 |
| 9 | depth_CHLmax + MLD + isolume | 365.09 | 16.16 | 0.00 | 0.71 ± 0.0027 | 92.64 ± 0.056 | 42.02 ± 0.58 | 99.37 ± 0.063 | 0.54 ± 0.0047 |
| 10 | depth_CHLmax + density_MLD + MLD + isolume | 365.52 | 16.58 | 0.00 | 0.71 ± 0.0038 | 92.69 ± 0.092 | 42.36 ± 0.76 | 99.39 ± 0.047 | 0.54 ± 0.0074 |
| 11 | depth_CHLmax + density_MLD + integrated_chl + isolume + thermocline | 350.93 | 2.00 | 0.07 | 0.72 ± 0.0041 | 92.59 ± 0.14 | 45.51 ± 0.79 | 98.85 ± 0.1 | 0.55 ± 0.0087 |
| 12 | depth_CHLmax + density_MLD + MLD + temp_above + integrated_chl + isolume + surface_PAR | 351.25 | 2.32 | 0.06 | 0.73 ± 0.0054 | 92.78 ± 0.17 | 46.74 ± 1.1 | 98.91 ± 0.12 | 0.57 ± 0.0011 |
| 13 | depth_CHLmax + density_MLD + MLD + thermocline + integrated_chl + isolume + surface_PAR | 351.83 | 2.89 | 0.05 | 0.73 ± 0.0037 | 92.72 ± 0.17 | 47.64 ± 0.79 | 98.71 ± 0.2 | 0.57 ± 0.0084 |
| 14 | depth_CHLmax + integrated_chl + isolume + thermocline | 351.92 | 2.99 | 0.04 | 0.72 ± 0.0048 | 92.45 ± 0.15 | 44.16 ± 0.93 | 98.88 ± 0.079 | 0.54 ± 0.01 |
| 15 | depth_CHLmax + density_MLD + MLD + thermocline + temp_below + integrated_chl + isolume + surface_PAR | 353.49 | 4.56 | 0.02 | 0.73 ± 0.0046 | 92.63 ± 0.21 | 47.3 ± 0.83 | 98.65 ± 0.17 | 0.56 ± 0.011 |
| 16 | depth_CHLmax + density_MLD + MLD + integrated_chl + isolume + CHLmax + surface_PAR | 355.29 | 6.36 | 0.01 | 0.73 ± 0.0056 | 92.64 ± 0.29 | 47.3 ± 0.98 | 98.67 ± 0.25 | 0.56 ± 0.015 |
| 17 | depth_CHLmax + density_MLD + isolume + CHLmax | 355.97 | 7.04 | 0.01 | 0.71 ± 0.0023 | 92.59 ± 0.2 | 42.92 ± 0.47 | 99.19 ± 0.25 | 0.54 ± 0.0083 |
| 18 | depth_CHLmax + density_MLD + integrated_chl + surface_PAR | 357.38 | 8.45 | 0.00 | 0.72 ± 0.0043 | 92.64 ± 0.12 | 45.17 ± 0.89 | 98.95 ± 0.12 | 0.55 ± 0.0081 |
| 19 | depth_CHLmax + density_MLD + MLD + integrated_chl + surface_PAR | 357.92 | 8.99 | 0.00 | 0.72 ± 0.0029 | 92.73 ± 0.12 | 45.06 ± 0.64 | 99.07 ± 0.15 | 0.56 ± 0.0057 |
| ***Diffuse Aggregations*** | |  |  |  |  |  |  |  |  |
| 1 | **depth_CHLmax + integrated_chl + isolume** | 364.20 | **0.00** | 0.16 | 0.76 ± 0.0056 | 92.43 ± 0.14 | 54.6 ± 1.1 | 97.33 ± 0.047 | 0.58 ± 0.0098 |
| 2 | **depth_CHLmax + integrated_chl + isolume + temp_above** | 364.23 | **0.03** | 0.16 | 0.76 ± 0.0027 | 92.35 ± 0.062 | 54.02 ± 0.54 | 97.32 ± 0 | 0.58 ± 0.0046 |
| 3 | **depth_CHLmax + density_MLD + integrated_chl + isolume** | 364.96 | **0.76** | 0.11 | 0.76 ± 0.0025 | 92.47 ± 0.075 | 54.94 ± 0.48 | 97.33 ± 0.047 | 0.59 ± 0.0046 |
| 4 | **depth_CHLmax + density_MLD + MLD + integrated_chl + isolume** | 365.59 | **1.39** | 0.08 | 0.76 ± 0.0039 | 92.47 ± 0.097 | 54.71 ± 0.8 | 97.36 ± 0.072 | 0.58 ± 0.0066 |
| 5 | **depth_CHLmax + integrated_chl + isolume + thermocline** | 365.67 | **1.47** | 0.08 | 0.76 ± 0.0027 | 92.35 ± 0.062 | 54.02 ± 0.54 | 97.32 ± 0 | 0.58 ± 0.0046 |
| 6 | **depth_CHLmax + isolume** | 365.69 | **1.49** | 0.08 | 0.76 ± 0.00 | 92.48 ± 0 | 55.17 ± 0 | 97.32 ± 0 | 0.59 ± 0.00 |
| 7 | **depth_CHLmax + density_MLD + temp_above + integrated_chl + isolume** | 365.91 | **1.71** | 0.07 | 0.76 ± 0.0018 | 92.32 ± 0.056 | 53.91 ± 0.36 | 97.3 ± 0.047 | 0.58 ± 0.0033 |
| 8 | depth_CHLmax + density_MLD + integrated_chl + isolume + thermocline | 366.81 | 2.61 | 0.04 | 0.76 ± 0.0030 | 92.41 ± 0.07 | 54.6 ± 0.61 | 97.32 ± 0 | 0.58 ± 0.0051 |
| 9 | depth_CHLmax + density_MLD + MLD + integrated_chl + isolume + surface_PAR | 367.47 | 3.27 | 0.03 | 0.76 ± 0.41 | 92.47 ± 0.12 | 54.6 ± 0.81 | 97.38 ± 0.077 | 0.58 ± 0.0073 |
| 10 | depth_CHLmax + MLD + isolume | 367.59 | 3.39 | 0.03 | 0.76 ± 0.00 | 92.48 ± 0 | 55.17 ± 0 | 97.32 ± 0 | 0.59 ± 0.00 |
| 11 | depth_CHLmax + density_MLD + MLD + isolume | 367.90 | 3.70 | 0.03 | 0.76 ± 0.0018 | 92.47 ± 0.042 | 55.06 ± 0.36 | 97.32 ± 0 | 0.59 ± 0.003 |
| 12 | depth_CHLmax + density_MLD + isolume + CHLmax | 367.96 | 3.76 | 0.02 | 0.76 ± 0.00 | 92.48 ± 0 | 55.17 ± 0 | 97.32 ± 0 | 0.59 ± 0.00 |
| 13 | depth_CHLmax + density_MLD + MLD + temp_above + integrated_chl + isolume + surface_PAR | 368.15 | 3.95 | 0.02 | 0.76 ± 0.0035 | 92.34 ± 0.075 | 53.79 ± 0.73 | 97.33 ± 0.047 | 0.58 ± 0.0056 |
| 14 | depth_CHLmax + MLD + surface_PAR | 368.38 | 4.18 | 0.02 | 0.76 ± 0.00 | 92.48 ± 0 | 55.17 ± 0 | 97.32 ± 0 | 0.59 ± 0.00 |
| 15 | depth_CHLmax + density_MLD + integrated_chl + surface_PAR | 368.56 | 4.36 | 0.02 | 0.76 ± 0.0028 | 92.44 ± 0.064 | 54.83 ± 0.56 | 97.32 ± 0 | 0.58 ± 0.0046 |
| 16 | depth_CHLmax + density_MLD + MLD + integrated_chl + surface_PAR | 368.83 | 4.63 | 0.02 | 0.76 ± 0.0033 | 92.34 ± 0.075 | 53.91 ± 0.65 | 97.32 ± 0 | 0.58 ± 0.0055 |
| 17 | depth_CHLmax + density_MLD + MLD + thermocline + integrated_chl + isolume + surface_PAR | 368.88 | 4.68 | 0.02 | 0.76 ± 0.0063 | 92.32 ± 0.16 | 53.91 ± 1.3 | 97.3 ± 0.085 | 0.58 ± 0.011 |
| 18 | depth_CHLmax + density_MLD + MLD + thermocline + temp_below + integrated_chl + isolume + surface_PAR | 369.10 | 4.90 | 0.01 | 0.75 ± 0.0064 | 92.24 ± 0.16 | 52.99 ± 1.3 | 97.33 ± 0.047 | 0.57 ± 0.011 |
| 19 | depth_CHLmax + density_MLD + MLD + integrated_chl + isolume + CHLmax + surface_PAR | 371.10 | 6.90 | 0.01 | 0.75 ± 0.0038 | 92.31 ± 0.13 | 53.1 ± 0.73 | 97.39 ± 0.079 | 0.57 ± 0.0075 |

**Supplementary Table S2.** REMUS and aggregation sampling information. The number of dense and diffuse aggregations detected by the REMUS in comparison to total number of profiles sampled.

| **Day of Year** | **# of Dense Aggregations** | **# of Profiles with Dense Aggregations** | **# of Diffuse Aggregations** | **# of Profiles with Diffuse Aggregations** | **Total Profiles** |
| --- | --- | --- | --- | --- | --- |
| 12 | 12 | 8 | 51 | 24 | 33 |
| 17 | 7 | 5 | 4 | 1 | 35 |
| 18 | 1 | 0 | 2 | 0 | 81 |
| 19 | 4 | 4 | 4 | 3 | 78 |
| 20 | 19 | 14 | 36 | 9 | 77 |
| 21 | 75 | 37 | 89 | 30 | 41 |
| 24 | 6 | 4 | 7 | 6 | 79 |
| 25 | 23 | 18 | 30 | 19 | 103 |
| 27 | 0 | 0 | 1 | 0 | 89 |
| 30 | 0 | 0 | 0 | 0 | 75 |
| 31 | 1 | 1 | 2 | 1 | 87 |
| TOTAL: | 148 | 91 | 226 | 93 | 778 |

Due to missing data or multiple aggregations detected in one profile, the number of profiles with dense and diffuse aggregations is often less than the number aggregations detected.


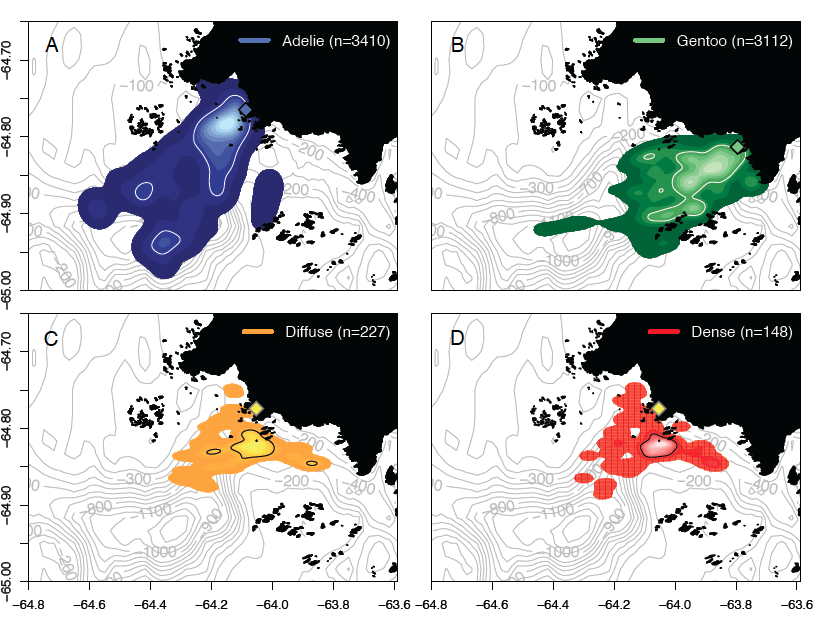


**Supplementary Figure S1**. Kernel density estimates of foraging locations for (A) Adélie penguins breeding on Humble Island (blue diamond), (B) gentoo penguins breeding on Biscoe Point (green diamond), and (C) diffuse and (D) dense aggregations detected acoustically by the REMUS near Palmer Station (yellow diamond). The white or black 50% contour lines represent the core foraging areas of penguins, and the primary area with aggregation detections. The maps were produced in R (R Development Core Team 2014).


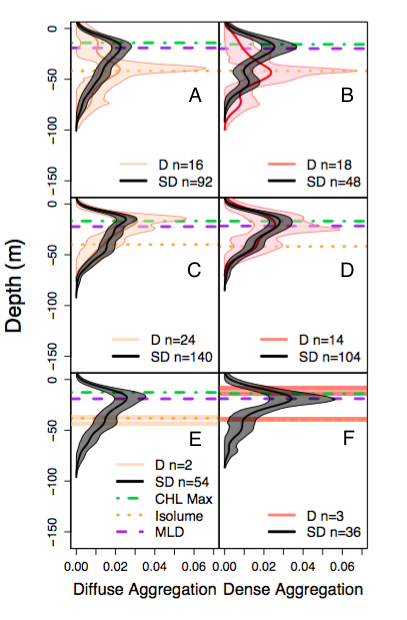


**Supplemental Figure S2**. During diurnal (D) and semidiurnal (SD) tides, we measured physical and biological properties within the Adélie penguin foraging habitat (top panel), gentoo penguin foraging habitat (middle panel) and the overlapping region where both species foraged (bottom panel). The horizontal lines represent the mean depth of the CHL_max_, the 1 W/m^2^ isolume and mixed layer depth (MLD) within profiles with diffuse or dense aggregations and within each respective penguin foraging contour (Fig. 1B,D). The kernel density estimate of the depth of (A) diffuse and (B) dense aggregations within the Adélie foraging habitat, (C) diffuse and (D) dense aggregations within the gentoo foraging habitat, and (E) diffuse and (F) dense aggregations within the overlapping region where both species forage. Due to small sample size during diurnal tides, the depth of aggregations are shown as horizontal lines in EF. The 95% confidence interval is shown around each kernel density estimate. Sample size (n) in each panel represents the number of aggregations during each respective tidal regime.


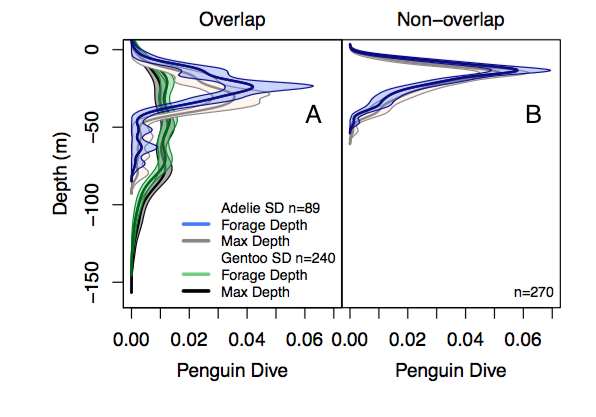


**Supplemental Figure S3**. Comparison of Adelie dive depth distributions in areas that overlap and don't overlap with gentoo penguins during semidiurnal tides. (A) The kernel density estimate foraging and maximum dive depths within the overlapping region of the gentoo foraging habitat (Adélie n=1, gentoo n=3). (B) The kernel density estimate of the Adélie penguin dive depths in the area of non-overlap. The 95% confidence interval is shown around each kernel density estimate. Sample size (n) represents the number of penguin dives.

***
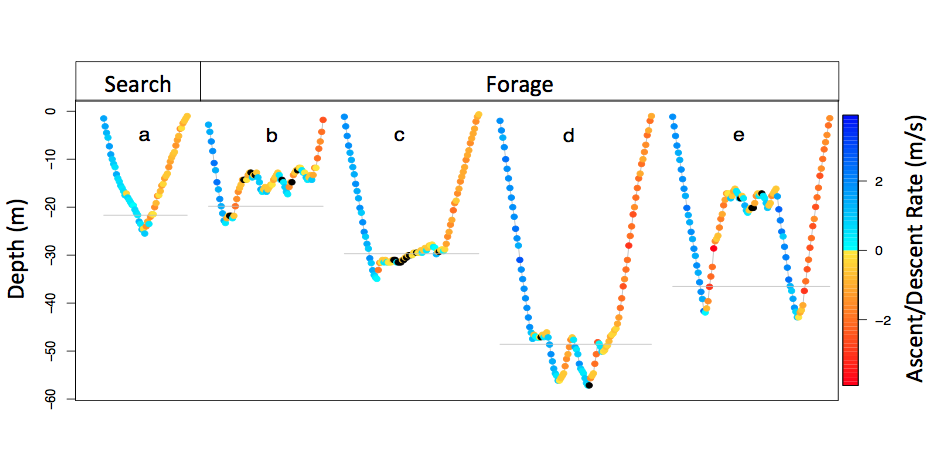
***

**Supplementary Figure S4.** Comparison of search and foraging dive behaviors. Foraging behaviors include plateaus (b, e), wiggles (b, d, e), or bottom time (c, d). The gray horizontal line represents the ledge or 85% of the maximum depth for each dive.

**Supplementary Figure S5.** Comparison of Chlorophyll (CHL, green) and acoustic scattering (black) profiles with depth (R = 0.96). This example shows the high correlation between CHL and acoustic scattering for a profile where an aggregation was not detected.

**Supplemental Figure S6.** Similar krill size class frequency distributions between Adélie and gentoo penguin diet samples during 2011.
